# Societá Italiana de Fisioterapia and the Physiotherapy Evidence Database (PEDro)

**DOI:** 10.1186/s40945-019-0058-3

**Published:** 2019-03-14

**Authors:** Mark R. Elkins, Anne M. Moseley

**Affiliations:** 1 The University of Sydney, Faculty of Medicine and Health, Sydney School of Public Health, Institute for Musculoskeletal Health, Sydney, Australia; 2 Centre for Education and Workforce Development, Sydney Local Health District, Building 301, Rozelle, 2039 Australia

**Keywords:** Physiotherapy, Evidence-based practice, PEDro, Professional issues

## Abstract

This paper provides an overview of a free resource that can be used by physiotherapists to assist their efforts to undertake evidence-based practice. The resource is the Physiotherapy Evidence Database (PEDro; www.pedro.org.au) – a searchable online database that in February 2019 indexes the details of over 42,000 pieces of published evidence about the effects of physiotherapy interventions. PEDro is searched millions of times each year by users worldwide. Societá Italiana de Fisioterapia (SIF; www.sif-fisioterapia.it) has entered into a collaboration with the developers of PEDro. In addition to describing the evidence available on PEDro and who uses it, this paper also summarises the features of PEDro that can facilitate evidence-based physiotherapy. This paper concludes by outlining the collaboration between SIF and PEDro.

## Evidence-based physiotherapy

The approach to the clinical care of patients known as “evidence-based practice” is becoming more widely accepted within the physiotherapy profession. The approach was defined by its developers as the “integration of the best research evidence with clinical expertise and patient values” [1]. Clinical physiotherapists who want their practice to be evidence-based must therefore identify the best evidence that is available to help inform their decisions about patient management.

It is difficult for physiotherapists to keep abreast of all the research that might be relevant to the types of patients they treat in clinical practice. One contributor to this difficulty is that, with ongoing publications, the number of trials of physiotherapy interventions is growing exponentially [2, 3]. If we consider physiotherapists who graduated in 2011, their university training could only have been based on about half of the evidence that currently exists about the efficacy of physiotherapy interventions. Another issue is that it can be laborious to find the relevant evidence on databases. For example, if a physiotherapist wanted to find evidence about the effects of physiotherapy treatments for knee osteoarthritis, a search of ‘knee osteoarthritis’ on the PubMed database in February 2019 returned over 31,500 articles, many of which have nothing to do with physiotherapy interventions. Searching can be targeted towards more relevant articles but this requires a knowledge of sophisticated search strategies, which involve category searches, Medical Subject Headings (MeSH) terms, Boolean operators, truncation and quotations [4, 5]. This inefficiency is an important issue because most clinical physiotherapists have limited time to find and read evidence. It would be simpler and more efficient if physiotherapists seeking evidence to guide their clinical practice could use a database that indexed only research publications about the effects of physiotherapy interventions.

## Physiotherapy Evidence Database (PEDro)

To address the situation described above, a group of physiotherapists established the Physiotherapy Evidence Database. More commonly referred to as ‘PEDro’, the database is freely available for anyone to use at www.pedro.org.au. This section of the paper will describe the content and features of PEDro, relating these to how they can assist physiotherapists who want to keep abreast of the growing body of evidence about physiotherapy interventions. This section will conclude with a review of how often and how widely PEDro is used.

### Content of PEDro

#### Evidence indexed on PEDro

PEDro indexes the bibliographic details and abstracts of three types of documents. One type of document is *randomised clinical trials* of physiotherapy interventions (or interventions that could become part of physiotherapy care). Another type of document is *systematic reviews* that include at least one randomised trial of a physiotherapy intervention.^1^ The third type of document is *clinical practice guidelines* that are based on a systematic literature search and that contain at least one recommendation relevant to physiotherapy practice. Although there are other forms of evidence (for example, inception cohort studies provide evidence about prognosis), the most unbiased evidence about the effects of interventions comes from the forms of evidence indexed on PEDro: randomised trials, systematic reviews and clinical practice guidelines.

**Fig. 1:**
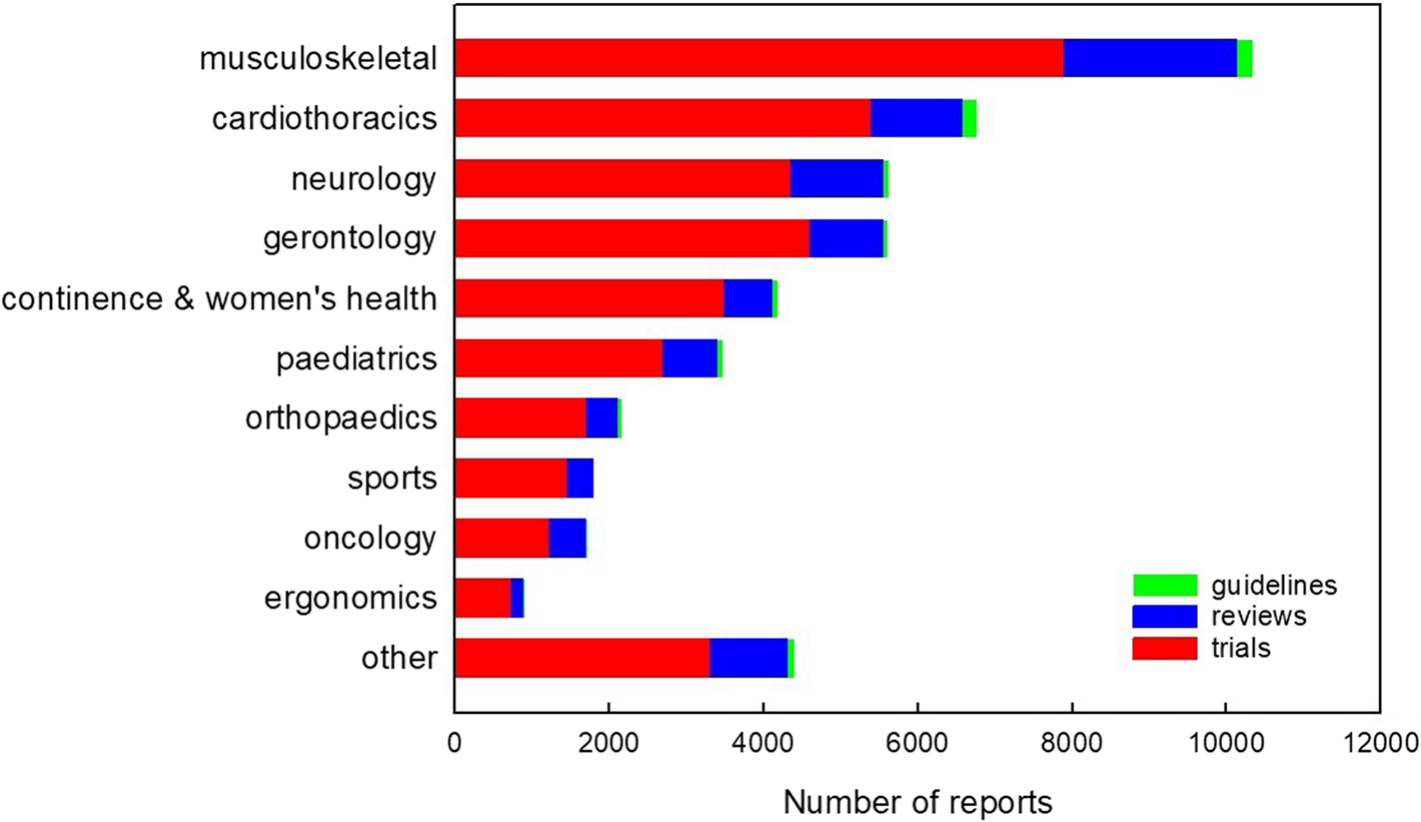
Amount of evidence indexed on PEDro, separated by subdiscipline of physiotherapy. Reproduced from https://www.pedro.org.au/english/downloads/pedro-statistics/

In February 2019, PEDro indexed over 33,000 trials, over 8000 systematic reviews, and over 650 clinical practice guidelines. The trials examine interventions from a wide range of subdisciplines, as shown in Fig. 1. This figure illustrates that the subdisciplines *musculoskeletal*, *cardiothoracics*, *neurology* and *gerontology* contribute the greatest share of records to PEDro, although even the subdisciplines with the fewest records have substantial evidence for interested users.

#### Growth in the content available on PEDro

**Fig. 2:**
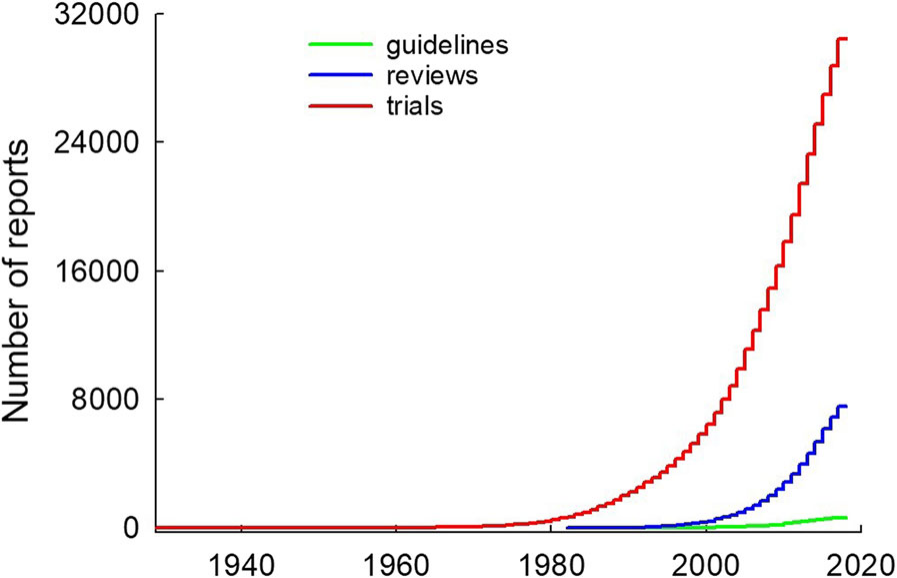
Cumulative number of trials, reviews and guidelines indexed on PEDro. Reproduced from https://www.pedro.org.au/english/downloads/pedro-statistics/

**Fig. 3:**
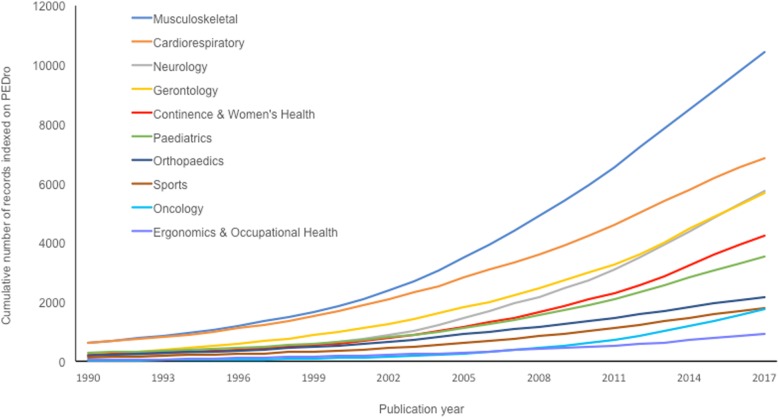
Cumulative number of records indexed on PEDro, separated by subdiscipline of physiotherapy based on June 2018 update

In 2010 and again in 2014, it was reported that the number of records indexed on PEDro was expanding exponentially [2, 3]. This pattern of rapid growth has continued and is shown in Fig. 2. The contribution of each subdiscipline to the growth in the content of PEDro is shown in Fig. 3.

The sustained exponential growth in research into the effects of physiotherapy interventions generates a wonderful body of evidence for the profession to draw upon. However, it also portrays the growing difficulty that a physiotherapist, whether working clinically or in academia, would have in keeping abreast of the evidence relevant to their areas of interest.

#### Completeness of coverage of evidence by PEDro

Two studies have examined the completeness of coverage of randomised trials of physiotherapy interventions by PEDro and a range of other databases [4, 5]. Although different methods were used in the two studies, the results were remarkably consistent. In both studies, PEDro and CENTRAL (which is the Cochrane Collaboration’s database of randomised trials) were ranked the top two databases, with respect to coverage of randomised trials of physiotherapy interventions. PEDro’s coverage was estimated at 92 and 99% in the two studies. Other databases, such as Embase and CINAHL, were ranked closely behind PEDro and CENTRAL. Given that PEDro is one of several databases that have good coverage of randomised trials of physiotherapy interventions, the next section of this paper will consider the features of PEDro that make it particularly useful and efficient for physiotherapists.

### Features of PEDro

#### Only evidence about physiotherapy interventions is indexed

As discussed at the beginning of this paper, searching a general database for high-quality evidence about physiotherapy interventions is inefficient. Even if the desired study design (eg, randomised trial, systematic review) is successfully incorporated into the search terms, it is still likely that many trials or reviews related to surgery, medication or other non-physiotherapy interventions will be retrieved. Because PEDro only indexes randomised trials, systematic reviews and clinical practice guidelines related to physiotherapy interventions, it permits a more targeted and therefore more efficient search for physiotherapists who want to know about the effects of an intervention.

#### Features of the searching process and search results

In addition to targeting evidence about physiotherapy interventions, searches of PEDro are designed to be efficient in other ways. Searches can specify the type of therapy from a pull-down menu that includes 13 categories. These categories (in order of the amount of evidence available on PEDro) are: fitness training; strength training; education; stretching, mobilisation, manipulation or massage; skill training; behaviour modification; electrotherapy, heat or cold; acupuncture; respiratory therapy; orthoses, taping or splinting; health promotion; neurodevelopmental therapy or neurofacilitation; and hydrotherapy or balneotherapy. In addition, searches can specify the problem experienced by the patient from a pull-down menu that includes 12 categories. These categories (in order of the amount of evidence available on PEDro) are: pain; reduced exercise tolerance; muscle weakness; motor incoordination; impaired ventilation; muscle shortening or reduced joint compliance; frailty; incontinence; oedema; reduced work tolerance; difficulty with sputum retention; and skin lesion, wound or burn. The search can also be targeted to one of 11 body regions or one of the 10 subdisciplines shown in Fig. 3. Users can specify as few or as many of these search fields as they wish. Users can also enter their own search terms by entering free text to be sought in the title and abstract.

If a particular known paper is required, then PEDro can also be searched by entering whichever of the following citation details are known: journal, year of publication, author, and words in the title. In fact, any combination of the search fields that have been mentioned so far can be used in a search.

The study design of the desired evidence (randomised trial, systematic review or clinical practice guideline) is another field that can be specified from a pull-down menu. If none of these is selected, all types of evidence will be retrieved, with clinical practice guidelines (if any are available) listed first in the search results, because they are the most condensed form of evidence. Next will be listed any systematic reviews that are identified by the search, with Cochrane reviews listed first because on average they use more rigorous methods than non-Cochrane reviews [6]. Last in the list of search results will be any relevant randomised trials, ordered from highest to lowest quality based on the PEDro Scale score [7]. The PEDro Scale includes items related to methodological quality and completeness of statistical reporting, resulting in a score out of 10 (with 0/10 being low and 10/10 being high quality) [7]. Several studies have reported acceptably high reliability for individual ratings and consensus ratings of both the English [7] and Portuguese [8] versions of the PEDro Scale. A Rasch analysis of the PEDro Scale [9] has provided evidence that the PEDro Scale can be used as a continuous scale for measuring the methodological quality and statistical reporting of trials. There is also evidence for convergent and construct validity of the PEDro Scale summary score and 8 of the 11 individual items [10]. The PEDro Scale has also been translated into Italian (https://www.pedro.org.au/italian/downloads/pedro-scale/).

The order in which search results are listed on the site is associated with how often the indexed research papers are accessed by users of PEDro. Specifically, synthesis research (ie, guidelines and reviews) are more commonly accessed than trials; Cochrane reviews are more commonly accessed than other reviews; and trials with higher PEDro Scale summary scores are more commonly accessed than lower quality trials [11]. It is unclear, however, whether this is because users prefer evidence that is more condensed and higher quality, or because they are influenced by the presentation of the search results.

When users of PEDro run a search, the results are initially presented as a list of titles. If a user sees a paper listed in the search results that might be of interest, clicking on it will reveal further details, such as the abstract (subject to copyright approval from the publisher), the full details of which PEDro Scale items were achieved (for randomised trials only), and links to full-text sources of the paper. In a random sample of 100 papers indexed on PEDro, 100% had one or more links to a full-text version online and 46% had at least one link to a free full-text version of the paper. Users of PEDro also have the option to select records from the search results and to email themselves the list of selected records.

#### Facilitating use of PEDro by particular groups of users

The developers of PEDro have studied how users search the database [11], which revealed several common errors. This has led to many improvements in the user interface to warn users when they are making a search error. For example, if a user tries to use brackets to group words as part of a free-text search string, they will receive a warning message to point out that brackets cannot be used in this way. Another initiative in response to the study of how users search PEDro is the addition of a series of “how to” training videos to the PEDro Search Help page (https://www.pedro.org.au/italian/search-help/) to help users improve their search skills. These videos include how to: ask a clinical question in PICO format, perform a PEDro search, optimise searching, and access full-text copies of papers identified by the search. Most of these videos are available in 12 different languages, including Italian. In fact, the whole PEDro website is now available in Italian and 11 other languages.

In addition to searching PEDro to answer clinical questions, users can browse the latest high-quality clinical research in their area of practice using PEDro’s *Evidence in your inbox* service (https://www.pedro.org.au/italian/evidence-in-your-inbox/). Each month, the details of any trials, reviews and guidelines that have just been added to the database can be emailed to you for free. Simply choose from 15 areas of practice (ie, the 10 subdisciplines of physiotherapy shown in Fig. 1 plus cerebral palsy, chronic pain, chronic respiratory disease, neurotrauma, or whiplash) and details of the new records related to your area(s) of interest will be automatically sent to the email address you nominate. The email address you enter will not used for any other purposes.

### Usage of PEDro

#### International usage of PEDro

Some detailed studies of PEDro usage in individual countries (eg, Brazil [12], Japan [13]) have been conducted, which reveal interesting insights into regional disparities that may suggest different uptake of evidence-based practice by physiotherapists in different parts of those countries. A detailed regional analysis has not yet been conducted for Italy. However, some comments can be made about usage of PEDro in Italy. Although usage fluctuates in all countries, Italy usually accounts for about 3% of worldwide usage of PEDro as judged by the number of searches. Italy usually ranks between 5th and 10th highest when countries are ranked by absolute number of searches. However, on a per-capita basis, Italy has lower usage than some larger countries (such as Brazil) and some smaller countries (such as Netherlands and Australia).

#### Growth in usage of PEDro

**Fig. 4:**
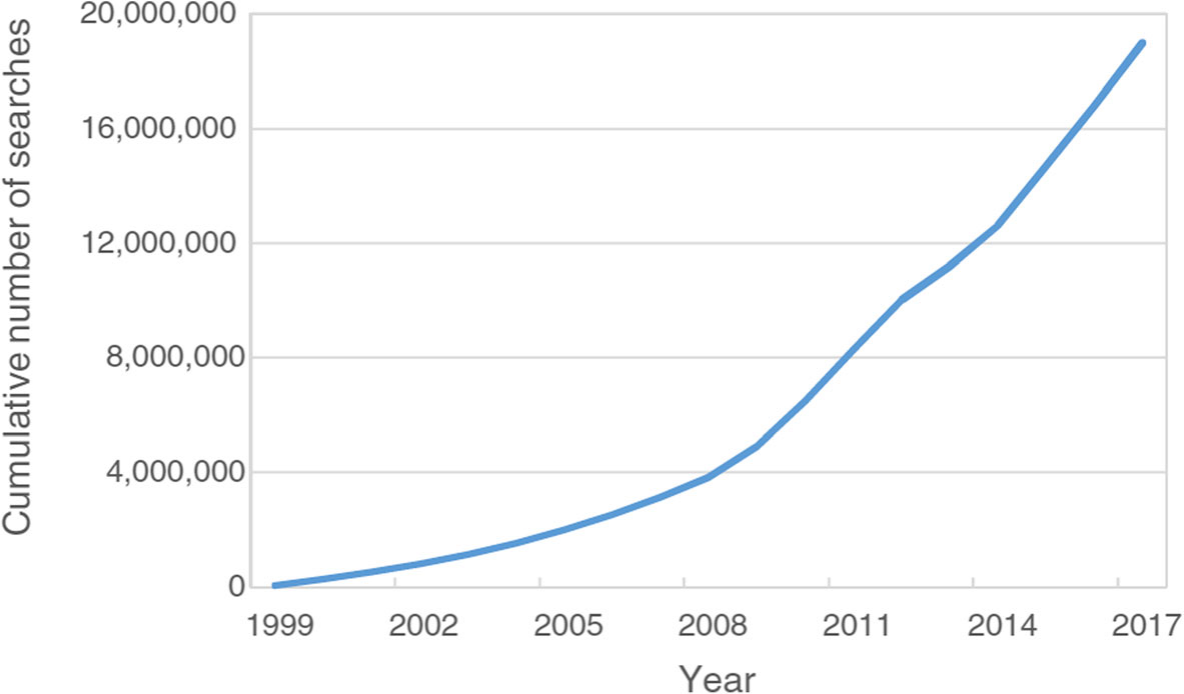
Cumulative number of searches of PEDro since its inception. Reproduced from Reference [14]

Since being launched in October 1999, PEDro has been used to answer 18,632,434 clinical questions [14]. The usage of PEDro has increased exponentially over time, as shown in Fig. 4. PEDro is now searched by users from virtually every country in the world, with an average of one search every 14 s. PEDro is increasingly searched as a source of trials for inclusion in systematic reviews. Over the past ten years, the number of systematic reviews indexed on the Cochrane Library that included a search of the PEDro database has increased exponentially [3].

## Collaboration between SIF and PEDro

SIF and its members make important contributions to PEDro. Along with 46 other physiotherapy associations and societies from around the world and other industry partners, SIF provides PEDro with some financial support. Collectively, these donations make it possible for PEDro to be kept up-to-date and freely available for the global physiotherapy community. Through this partnership with PEDro, SIF encourages its members to enhance their skills in aspects of evidence-based practice and actively contribute to the indexing of evidence in PEDro. Approximately 40 SIF members have completed the PEDro Scale Training Program (https://training.pedro.org.au/). This online course develops skills in appraising the methodological quality of randomised controlled trials.

Many SIF members are “friends of PEDro”, volunteering their time to rate trials using the PEDro Scale. The PEDro Scale scores displayed in PEDro are generated by teams of two or three raters, with two raters independently evaluating a trial and a third rater arbitrating any disagreements. In addition to rating the Italian-language trials indexed in PEDro (there were 53 Italian trials in the February 2019 update of PEDro), SIF members also rate trials in their area of clinical interest that are written in English. Collectively, SIF members have rated over 400 of the trials indexed on PEDro.

## Conclusion

By supporting and collaborating with PEDro, SIF is doing a lot to encourage physiotherapists to undertake evidence-based practice. Italian physiotherapists should respond by taking the opportunity to use the Italian-language interface to access PEDro to improve their knowledge of available evidence about physiotherapy interventions. Italian physiotherapists can be proud of the work that SIF has done to facilitate evidence-based physiotherapy, because it provides a great example for other national physiotherapy associations globally. If physiotherapy associations and societies in all countries followed this lead, patient care would be enhanced through the shared use of the best evidence.

## Abbreviations

CENTRAL: Cochrane Central Register of Controlled Trials
CINAHL: Cumulative Index to Nursing & Allied Health Literature
MeSH: Medical subject headings
PEDro: Physiotherapy Evidence Database
SIF: Societá Italiana de Fisioterapia

## Footnotes

1 Although most systematic reviews on PEDro include randomised trials, PEDro also indexes systematic reviews of systematic reviews (sometimes called an *umbrella review* or an *overview of reviews*) and even systematic reviews of clinical practice guidelines.
